# Supplementary material for: Divergent Patterns and Trends in Breast Cancer Incidence, Mortality and Survival Among Older Women in Germany and the United States
Source: Cancers (Basel). 2020 Aug 26;12(9):2419. doi: 10.3390/cancers12092419 (PMC7565138; doi:10.3390/cancers12092419)
Supplement: Supplementary file 1 [file cancers-12-02419-s001.pdf]

cancers

MDPI

Article

Divergent Patterns and Trends in Breast Cancer Incidence, Mortality and Survival Among Older Women in Germany and the United States

Lina Jansen [1,*], Bernd Holleczek [2], Klaus Kraywinkel [3], Janick Weberpals [1], Chloé Charlotte Schröder [1], Andrea Eberle [4], Katharina Emrich [5], Hiltraud Kajüter [6], Alexander Katalinic [7], Joachim Kieschke [8], Alice Nennecke [9], Eunice Sirri [8], Jörg Heil [10], Andreas Schneeweiss [11] and Hermann Brenner [1,12,13] for the GEKID Cancer Survival Working Group

1 Division of Clinical Epidemiology and Aging Research, German Cancer Research Center (DKFZ), 69120 Heidelberg, Germany; janick.weberpals@gmail.com (J.W.); ccschroeder@gmx.de (C.C.S.); h.brenner@dkfz.de (H.B.)
2 Saarland Cancer Registry, 66119 Saarbrücken, Germany; B.Holleczek@gbe-ekr.saarland.de
3 German Centre for Cancer Registry Data (ZfKD), Robert Koch-Institute, 13353 Berlin, Germany; KraywinkelK@rki.de
4 Cancer Registry of Bremen, Leibniz Institute for Prevention Research and Epidemiology - BIPS, 28359 Bremen, Germany; eberle@leibniz-bips.de
5 Cancer Registry of Rhineland-Palatinate, Institute for Medical Biostatistics, Epidemiology and Informatics, University Medical Center, Johannes Gutenberg University Mainz, 55116 Mainz, Germany; emrich@krebsregister-rlp.de
6 Cancer Registry of North Rhine-Westphalia, 44801 Bochum, Germany; Hiltraud.Kajueter@krebsregister.nrw.de
7 Cancer Registry of Schleswig-Holstein, 23552 Lübeck, Germany; alexander.katalinic@uksh.de
8 Cancer Registry of Lower Saxony, 26121 Oldenburg, Germany; kieschke@offis-care.de (J.K.); eunice.sirri@offis-care.de (E.S.)
9 Hamburg Cancer Registry, 20539 Hamburg, Germany; Alice.Nennecke@bsg.hamburg.de
10 Department of Gynecology and Obstetrics, University Women's Clinic, 69120 Heidelberg, Germany; joerg.heil@med.uni-heidelberg.de
11 National Center for Tumor Diseases, Division Gynecologic Oncology, University Hospital and German Cancer Research Center, 69120 Heidelberg, Germany; andreas.schneeweiss@nct-heidelberg.de
12 Division of Preventive Oncology, German Cancer Research Center (DKFZ), and National Center for Tumor Diseases (NCT), 69120 Heidelberg, Germany
13 German Cancer Consortium (DKTK), German Cancer Research Center, 69120 Heidelberg, Germany
* Correspondence: l.jansen@dkfz.de

Supplementary Methods

As the proportion of patients with missing stage was high and changed over time (**Table S3**), multivariate imputation by chained equation (MICE) was conducted separately on the German and the US dataset.[1] Using 10 iterations, 15 imputed datasets were created using the MICE package in R.[1] The imputation model included the factors age, sequence number of the BC, year of diagnosis, ICD-10 code, stage, grade, vital status, the Nelson-Estimator as estimate for follow-up time[2] and, in Germany, region. Model convergence was checked graphically and stage distributions and survival estimates were compared before and after imputation (**Table S3 & S4**). The imputed datasets were used in all analyses that included stage information.

Table S1. Overview of included cancer registries.

	Underlying population in millions [a]	Years of diagnosis	Case number [b]	%DCO in 2004-2015
Bavaria[c]	6.59	2003-14	59,722	4.5
Brandenburg	2.46	1998-2015	31,370	2.5
Bremen	0.66	1998-2014	9,483	3.7

Hamburg	1.76	1998-2015	26,076	3.6
Lower Saxony	7.83	2003-15	88,025	7.6
Mecklenburg-Vorpommern	1.60	1998-2015	20,509	2.6
North Rhine-Westphalia[d]	2.61	1998-2015	37,089	6.7
Rhineland-Palatinate	4.01	1998-2015	55,099	7.8
Saarland	0.99	1974-2015	29,127	1.6
Saxony	4.06	1998-2015	55,158	2.2
Saxony-Anhalt	2.24	1998-2015	28,770	5.4
Schleswig-Holstein	2.83	1999-2015	43 732	8.7
Thüringen	2.16	1998-2015	28,374	3.4
Total Germany	39.8	1974-2015	512,534	5.2
United States / SEER-9[e]	29.0	1975-2015	671,095	0.6

DCO=Death certificate only or autopsy only cases [a] Underlying population in 2014 in Germany and 2010 in the United States[b] Case number for all included years of diagnosis after exclusion of death certificate only cases[c] Included regions: Schwaben: Aichbach Friedberg, Augsburg city, Augsburg district; Oberfranken: Bayreuth city, Bayreuth district, Forchheim; Mittelfranken: Erlangen Hochstadt, Erlangen, Nürnberg, Fürth; Oberpfalz: all districts; Oberbayern: all districts except Berechtsgardener Land, Mühldorf am Inn, Landberg am Lech, Eichstädt, Ingolstadt, Pfaffenhofen a.d. Ilm; Niederbayern: Landshut city, Landshut district[d] Included regions: administrative region (Regierungsbezirk) Münster[e] Included regions in SEER-9: Atlanta, Connecticut, Detroit, Hawaii, Iowa, New Mexico, San Francisco-Oakland, Seattle-Puget Sound, and Utah. −

Table S2. Classification of morphology groups.

Morphology group	Included ICD-0-3 morphology
Invasive carcinoma of no special type	8500
Pleomorphic carcinoma	8522
Invasive lobular carcinoma	8520
Tubular carcinoma	8211
Mucinous carcinoma	8480
Medullary carcinoma	8510
Invasive micropapillary carcinoma	8507
Metaplastic carcinoma of no special type	8575, 8570, 8572, 8070, 8032, 8571, 8575, 8982
Invasive papillary carcinoma	8503
Other / not specified	All other morphology codes

cancers

MDPI

Table S3. Stage distribution before and after imputation in Germany and the United States for 1998-2000 to 2013-15.

Factors			Period						Difference [a] in % units
Country	Imputation	Stage [b]	1998–2000	2001–03	2004–06	2007–09	2010–12	2013–15	
Germany	Before	I	34.0%	36.0%	36.1%	39.3%	40.4%	40.4%	+6.4
		II	46.6%	43.1%	39.2%	38.3%	38.1%	38.7%	−7.9
		III	10.9%	12.9%	17.0%	15.0%	13.9%	13.2%	+2.3
		IV	8.5%	8.0%	7.8%	7.4%	7.5%	7.7%	−0.8
		Unknown[d]	30.4%	26.5%	26.0%	19.2%	16.4%	18.0%	−12.4
	After[c]	I	32.6%	34.6%	34.9%	38%	39.1%	39.3%	+6.7
		II	45.3%	42.3%	39.3%	38.5%	38.6%	39.4%	−5.9
		III	11.5%	13.6%	16.9%	15.4%	14.4%	13.7%	+2.2
		IV	10.5%	9.5%	8.9%	8.1%	7.8%	7.6%	−2.9
United States	Before	I	50.8%	50.0%	49.9%	49.9%	49.9%	50.3%	−0.5
		II	32.0%	32.8%	33.1%	33.2%	33.7%	34.3%	+2.3
		III	12.9%	12.7%	12.2%	11.7%	11.0%	10.1%	−2.8
		IV	4.4%	4.5%	4.8%	5.2%	5.4%	5.3%	−0.9
		Unknown[d]	9.2%	7.2%	5.3%	5.0%	3.6%	3.1%	−6.1
	After	I	49.9%	49.5%	49.4%	49.5%	49.6%	50.1%	+0.2
		II	32.3%	33.0%	33.2%	33.3%	33.8%	34.4%	+2.1
		III	13.8%	13.5%	12.8%	12.2%	11.3%	10.4%	−3.4
		IV	4.0%	4.1%	4.6%	5.0%	5.3%	5.1%	+1.1

[a] Difference between distributions in 2013–15 and 1998–2000 [b] In Germany and the United States, 872 and 575 patients, respectively, with stage 0 were excluded [c] Shown are the proportions over all imputation datasets [d] Proportion of patients with missing stage information among all patients.

Table S4. Five–year age–standardized stage–specific survival (standard error) before and after imputation in Germany and the United States for 2002–04 to 2011–13.

Factor			Year of diagnosis				Difference [a]
**Country**	**Imputation**	**Group**	**2004–06**	**2007–09**	**2010–12**	**2013–15**	
Germany	Before	Stage I	101.0 (0.4)	101.8 (0.3)	102.1 (0.3)	101.9 (0.3)	+0.9
		Stage II	93.0 (0.4)	94.6 (0.3)	94.3 (0.3)	94.7 (0.3)	+1.7
		Stage III	72.3 (0.8)	74.8 (0.6)	75.2 (0.5)	76.2 (0.5)	+3.9
		Stage IV	30.8 (0.8)	30.8 (0.7)	30.1 (0.6)	30.5 (0.6)	−0.3
		Unknown	76.8 (0.4)	76.2 (0.4)	75.7 (0.4)	78.6 (0.4)	+1.8
	After	Stage I	99.6 (0.4)	100.3 (0.3)	100.7 (0.3)	100.9 (0.3)	+1.3
		Stage II	89.9 (0.3)	91.1 (0.3)	91.4 (0.3)	92.2 (0.3)	+2.3
		Stage III	69.0 (0.7)	71.1 (0.6)	71.3 (0.5)	72.6 (0.5)	+3.6
		Stage IV	31.3 (0.8)	30.5 (0.7)	29.7 (0.6)	30.9 (0.6)	−0.4
United States	Before	Stage I	100.2 (0.3)	101.2 (0.3)	101.7 (0.3)	101.8 (0.3)	+1.6
		Stage II	90.1 (0.4)	90.7 (0.4)	92.1 (0.4)	92.0 (0.4)	+1.9
		Stage III	66.6 (0.8)	69.6 (0.8)	70.4 (0.8)	70.8 (0.8)	+4.2
		Stage IV	21.6 (0.9)	22.3 (0.9)	23.5 (0.9)	24.6 (0.8)	+3.0
		Unknown	74.3 (0.9)	70.4 (1.1)	69.6 (1.1)	67.0 (1.2)	−7.3
	After	Stage I	99.6 (0.3)	100.4 (0.3)	100.9 (0.3)	101.1 (0.3)	+1.5
		Stage II	88.5 (0.4)	89.0 (0.4)	90.4 (0.4)	90.7 (0.4)	+2.2
		Stage III	63.1 (0.9)	66.2 (0.9)	67.9 (0.8)	68.6 (0.8)	+5.5
		Stage IV	24.6 (1.2)	23.8 (1.1)	24.0 (1.0)	24.4 (0.8)	−0.2

[a] Difference between 5–year relative survival in 2002–04 and 2013–15.

Table S5. Patient and tumor characteristics of patients with a first invasive breast cancer diagnosed in Germany and the United States in 1974–2015.

Factor	Germany [a]	United States
	N (%)[b]	N (%)[b,c]
**N**	512,534	671,095
**Excluded DCO cases**	33,102 (6.1)	4,847 (0.7)
**Age at diagnosis**		
15–29	2,152 (0.4)	3,990 (0.6)
30–39	19,125 (3.7)	35,226 (5.2)
40–44	28,811 (5.6)	44,856 (6.7)
45–49	45,847 (8.9)	64,887 (9.7)
50–54	54,665 (10.7)	72,650 (10.8)
55–59	56,672 (11.1)	76,563 (11.4)
60–64	66,780 (13.0)	80,830 (12.0)
65–69	70,606 (13.8)	79,703 (11.9)
70–74	57,418 (11.2)	71,119 (10.6)
75–79	50,162 (9.8)	61,005 (9.1)
80+	60,296 (11.8)	80,266 (12.0)
Mean (standard deviation)	62.8 (13.6)	61.7 (14.3)
**Morphology**		
Invasive carcinoma of no special type	360,949 (70.4)	473,295 (70.5)
Pleomorphic carcinoma	16,875 (3.3)	33,940 (5.1)
Invasive lobular carcinoma	65,384 (12.8)	53,896 (8.0)
Tubular carcinoma	6,522 (1.3)	7,071 (1.1)
Mucinous carcinoma	9,235 (1.8)	14,578 (2.2)
Medullary carcinoma	3,674 (0.7)	6,969 (1.0)
Invasive micropapillary carcinoma	392 (0.1)	924 (0.1)
Metaplastic carcinoma of no special type	1,346 (0.3)	1,598 (0.2)
Invasive papillary carcinoma	2,145 (0.4)	1,771 (0.3)
Other / not specified	46,012 (9.0)	77,053 (11.5)
**Grade**		
Well differentiated (I)	NA[d]	96,705 (14.4)
Moderately differentiated (II)	NA[d]	199,789 (29.8)
Poorly differentiated (III)	NA[d]	180,398 (26.9)
Unknown	NA[d]	194,203 (28.9)
**Tumor site (ICD–10 code C50.X)**		
Nipple and areola (0)	7,167 (1.4)	5,945 (0.9)
Central portion (1)	23,098 (4.5)	37,347 (5.6)
Upper–inner quadrant (2)	48,766 (9.5)	65,364 (9.7)
Lower–inner quadrant (3)	24,838 (4.8)	33,585 (5.0)
Upper–outer quadrant (4)	167,487 (32.7)	224,881 (33.5)
Lower–outer quadrant (5)	34,673 (6.8)	44,611 (6.6)
Axillary tail (6)	971 (0.2)	5,118 (0.8)
Overlapping sites (8)	54,527 (10.6)	138,831 (20.7)
Unspecified site (9)	151,007 (29.5)	115,413 (17.2)
**Stage according to UICC/AJCC (1998–2015)[e]**		
I	184,229 (37.0)	181,151 (49.7)
II	199,018 (40.0)	121,719 (33.4)
III	72,508 (14.6)	44,665 (12.2)
IV	42,364 (8.5)	17,112 (4.7)

DCO=death certificate or autopsy only case; ICD=International Classification for Disease [a] For period of diagnosis 1974–1997, data was restricted to Saarland. [b] Percentage of the number of patients in each category in relation to the total number of patients in this country. Due to rounding, the

numbers might not add up to 100%. For age, the mean and standard deviation are shown in the line "mean (standard deviation)". [c] Data from the United States was only available starting in 1975. [d] Grade is not shown as the proportion of patients with missing information was too high in earlier years and decreased strongly over time (from > 90% in 1974–85 to < 10% in 2002–15). [e] Patients with stage 0 were excluded (N=872 (0.2%) (Germany) and N=603 (0.2%) (United States)).

Table S6. Five–year age–standardized and age–specific survival (standard error) in Germany and the United States in 1979–83 to 2013–15.

Factor		Year of diagnosis									Difference: 2013–15 to	
**Country**	**Age group**	**1979–83[b]**	**1984–88[b]**	**1989–93[b]**	**1994–98[b]**	**1999–03[b]**	**2004–06**	**2007–09**	**2010–12**	**2013–15**	**1979–83**	**2004–06**
Germany	All[a]	69.9 (1.6)	71.2 (1.4)	74.8 (1.2)	75.9 (1.1)	82.2 (0.3)	83.9 (0.2)	84.8 (0.2)	85.6 (0.2)	87.0 (0.2)	+17.1	+3.1
	50–69	67.5 (1.5)	69.9 (1.5)	74.7 (1.3)	77.5 (1.1)	84.8 (0.3)	87.2 (0.2)	88.6 (0.2)	90.8 (0.2)	91.9 (0.1)	+24.4	+4.7
	70+	67.9 (2.9)	71.0 (2.5)	73.7 (2.4)	74.4 (1.9)	78.7 (0.6)	79.3 (0.5)	79.6 (0.4)	79.3 (0.4)	81.1 (0.3)	+13.2	+1.8
United States	All[a]	71.9 (0.3)	75.2 (0.3)	81.3 (0.2)	83.8 (0.2)	86.9 (0.2)	88.4 (0.2)	89.2 (0.2)	90.0 (0.2)	90.4 (0.2)	+18.5	+2.0
	60–69	73.2 (0.3)	76.3 (0.3)	82.2 (0.2)	85.2 (0.2)	88.6 (0.2)	89.3 (0.2)	90.2 (0.2)	90.7 (0.2)	91.2 (0.2)	+18.0	+1.9
	70+	69.5 (0.6)	73.6 (0.5)	80.6 (0.4)	82.7 (0.4)	85.1 (0.4)	87.3 (0.5)	87.8 (0.5)	89.2 (0.5)	89.6 (0.5)	+20.1	+2.3

[a] Age–standardized [b] Estimates for Saarlan

cancers MDPI

Table S7. Five–year stage–specific survival and five–year age–standardized and age–specific survival (standard error) after standardization by stage in Germany and the United States in 2004–04 to 2013–15.

Factor			Year of diagnosis				Difference[a]
Country	Group	Standardization	2004–06	2007–09	2010–12	2013–15	
Germany	Stage I	Age	99.6 (0.4)	100.3 (0.3)	100.7 (0.3)	100.9 (0.3)	+1.3
	Stage II	Age	89.9 (0.3)	91.1 (0.3)	91.4 (0.3)	92.2 (0.3)	+2.3
	Stage III	Age	69.0 (0.7)	71.1 (0.6)	71.3 (0.5)	72.6 (0.5)	+3.6
	Stage IV	Age	31.3 (0.8)	30.5 (0.7)	29.7 (0.6)	30.9 (0.6)	−0.4
	All	Age & Stage	89.6 (0.2)	90.5 (0.2)	90.8 (0.2)	91.4 (0.2)	+1.8
	50–69	Stage	90.0 (0.2)	91.0 (0.1)	92.1 (0.1)	92.4 (0.1)	+2.4
	70+	Stage	88.9 (0.4)	89.6 (0.4)	89.1 (0.3)	89.9 (0.3)	+1.0
United States	Stage I	Age	99.6 (0.3)	100.4 (0.3)	100.9 (0.3)	101.1 (0.3)	+1.5
	Stage II	Age	88.5 (0.4)	89.0 (0.4)	90.4 (0.4)	90.7 (0.4)	+2.2
	Stage III	Age	63.1 (0.9)	66.2 (0.9)	67.9 (0.8)	68.6 (0.8)	+5.5
	Stage IV	Age	24.6 (1.2)	23.8 (1.1)	24.0 (1.0)	24.4 (0.8)	−0.2
	All	Age & Stage	88.1 (0.2)	89.0 (0.2)	90.0 (0.2)	90.2 (0.2)	+2.1
	50–69	Stage	89.1 (0.2)	90.1 (0.2)	90.7 (0.2)	91.1 (0.2)	+2.0
	70+	Stage	86.8 (0.4)	87.3 (0.5)	88.7 (0.4)	88.8 (0.4)	+2.0

[a] Difference between 5–year relative survival in 2013–15 and 2004–06 in percent units.

References

1. van Buuren S, Groothuis-Oudshoorn K: mice: Multivariate Imputation by Chained Equations in R. Journal of Statistical Software 45:1-67, 2011
2. White IR, Royston P: Imputing missing covariate values for the Cox model. Stat Med 28:1982-98, 2009
